# Proteomic analyses of decellularized porcine ovaries identified new matrisome proteins and spatial differences across and within ovarian compartments

**DOI:** 10.1038/s41598-019-56454-3

**Published:** 2019-12-27

**Authors:** Nathaniel F. Henning, Richard D. LeDuc, Kelly A. Even, Monica M. Laronda

**Affiliations:** 1 Department of Pediatrics, Feinberg School of Medicine, Northwestern University, Chicago, USA; 2 Stanley Manne Children’s Research Institute, Ann & Robert H. Lurie Children’s Hospital of Chicago, Chicago, USA; 3 Proteomics Center of Excellence, Northwestern University, Evanston, USA

**Keywords:** Proteomics, Tissue engineering

## Abstract

Premature ovarian insufficiency (POI) affects approximately 1% of women. We aim to understand the ovarian microenvironment, including the extracellular matrix (ECM) and associated proteins (matrisome), and its role in controlling folliculogenesis. We mapped the composition of the matrisome of porcine ovaries through the cortical compartment, where quiescent follicles reside and the medullary compartment, where the larger follicles grow and mature. To do this we sliced the ovaries, uniformly in two anatomical planes, enriched for matrisome proteins and performed bottom-up shotgun proteomic analyses. We identified 42 matrisome proteins that were significantly differentially expressed across depths, and 11 matrisome proteins that have not been identified in previous ovarian protein analyses. We validated these data for nine proteins and confirmed compartmental differences with a second processing method. Here we describe a processing and proteomic analysis pipeline that revealed spatial differences and matrisome protein candidates that may influence folliculogenesis.

## Introduction

Premature ovarian insufficiency (POI), or early menopause, is a serious off-target effect for approximately 1 in 6 female cancer survivors^1^. In the United States more than 11,000 children aged 0 to 14 will be diagnosed with cancer this year alone, resulting in approximately 1,800 new cases of gonadal insufficiency per year^1^. POI has systemic biological effects, beyond fertility, that result from the lack of ovarian hormones, and include an increased risk for several comorbidities^2–5^.

The ovary is divided into two visibly distinct compartments: the denser cortex, containing the pool of primordial follicles, or ovarian reserve, and the more permissive medulla containing the majority of activated and growing follicles^6,7^. After activation, follicles respond to soluble signals as well as physical stresses, created by mechanical forces and osmotic shifts, to grow, mature and ovulate (reviewed in^8^). It is hypothesized that the two compartments have varying rigidities and that during folliculogenesis the activated follicles migrate into the less rigid medullary compartment of the ovary. It is believed that passage down this rigidity gradient alters cellular responses of the follicle to hormones, which changes gene expression of the cells making up the follicle^9,10^. This is supported by analyzing health, growth and gene expression of isolated murine and primate follicles cultured in different biomaterials^11–15^. However, the relationship between the native extracellular environment or niche and ovarian follicle in the form of signal transduction cues to maintain quiescence or trigger activation continues to remain unclear.

Previous work has shown that a bioprosthetic ovary made with a 3D printed gelatin scaffold restores hormone production and fertility in ovariectomized mice^16^. To improve upon this work and move toward translation for human use, a clear understanding of the native environment and how scaffolding components can influence folliculogenesis is required. An appropriately functioning organ will require specific extracellular cues and understanding those cues will inform bioengineering for tissue regeneration. The matrisome provides the ultrastructure that supports the tissue-specific microenvironment *in vivo*^17^. There are several examples of three-dimensional ECM materials providing the ideal transplantable scaffold^7,18–21^. This is because such a scaffold provides the necessary signaling cues that control tissue-specific cell attachment, differentiation, vascularization, and functionality^22,23^.

Previous studies examined the ovarian matrisome of human cortical tissue or whole ovaries^24,25^. However, none have examined the differences between the two main compartments or considered the three-dimensional composition of the ovarian matrisome. The goal of this study was to spatially map the matrisome composition of the ovary in order to determine the ovarian cortical and medullary matrisome signatures using a proteomic-based approach on decellularized tissue. We chose to use porcine ovaries, as they have similar compartmentalization and folliculogenesis waves to human ovaries and can be processed in the same way^26–29^. This data and future functional assays will inform an improved bioprosthetic ovary with a better biomimetic scaffold.

## Materials and Methods

### Porcine ovary processing for protein analysis

**Figure 1:**
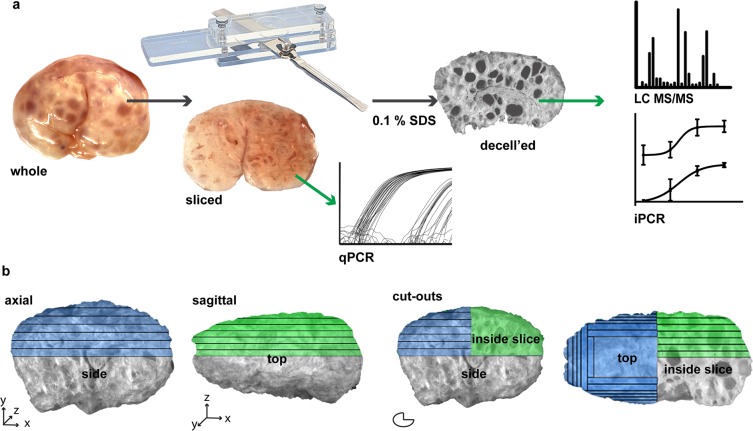
Schematic of (**a**) processing the porcine ovary with a tissue slicer, prior to qPCR analysis, decellularization then proteomics analysis and iPCR validation. (**b**) Ovaries were sliced axially and sagittally. SDS, sodium dodecyl sulfate; decell’ed, decellularized; LC MS/MS, liquid chromatography tandem mass spectrometry; iPCR, immuno PCR.

Porcine ovaries were purchased from Tissue Source, LLC. Pigs were peri-pubertal (4–6 months old) when sacrificed and ovaries retrieved. Ovaries were shipped overnight in PBS. Porcine ovaries were processed in two ways: **(1)** ***uniform slices:*** Upon arrival, ovaries were bisected sagittally through the hilum or axially into two equal halves (Fig. 1). Bisected pieces were further processed using a Stadie-Riggs slicer, which produces 0.5 mm slices (Fig. 1a). Slices were imaged using a pathology scope (Virtus Imaging, PX-XT-PC) and weighed. Sliced tissue was either flash frozen in liquid nitrogen for RNA extraction or proceeded through decellularization steps. **(2)** ***removal of cortex prior to slicing:*** Porcine ovaries were bisected sagitally as above. The first two slices (1 mm) of the ovary were taken and decellularized together as the cortical region. The remaining tissue was pinned and trimmed using a razor blade 1 mm in from the ovarian surface, leaving only medullary tissue. The medulla was then processed into 0.5 mm slices. Trimmed pieces (cortex) were taken whole for further processing as well. All slices were decellularized.

### Porcine ovary decellularization

Decellularization was carried out using 0.1% sodium dodecyl sulfate (SDS, Sigma, 75746) in phosphate buffered saline (PBS, Thermo, 10010023). Slices were placed on a nutator at 4 °C and SDS solution was changed every 24 hours for 48–72 hours prior to protein extraction. Several slices were set aside for DNA extraction (Zymo, D4075) and quantification. Extracted DNA was quantified using a spectrophotometer (MidSci NanoPhotometer, NP60). All slices contained less DNA than the recommended standard of 50 ng/mg of tissue^30^ and contained an average of 20.75 ng/mg.

### Sample preparation for liquid chromatography tandem mass spectrometry (LC-MS/MS) analysis

Two technical replicates of four porcine ovaries from four separate animals (two ovaries per direction) were used to generate seven slices each in the proteomics analysis. 50 µg of protein was precipitated with eight volumes of cold acetone (Fisher, A18–4) and one volume of trichloroacetic acid (Sigma, T9159-250G) overnight at −20 °C. After washing the pellet with ice-cold acetone, resulting protein pellet was resuspended in 50 µL 8 M urea (Invitrogen, 15505-035) in 400 mM ammonium bicarbonate (Fisher, A643-500), pH 7.8, reduced with 4 mM dithiothreitol (Sigma, 10197777001) at 50 °C for 30 min., and cysteines were alkylated with 18 mM iodoacetamide (Sigma, I1149) in the dark for 30 min. The solution was then diluted to <2 M urea and trypsin (Promega, V5280) was added at final trypsin:protein ratio of 1:50 prior to overnight incubation at 37 °C with shaking. The resulting peptides were desalted using solid phase extraction on a Pierce C18 Spin column (Thermo, 89873) and eluted in 80 mL of 80% acetonitrile (ACN) (Thermo, 51101) in 0.1% formic acid (FA) (Fisher, LS118). After lyophilization, peptides were reconstituted with 5% ACN in 0.1% FA.

### LC-MS/MS data acquisition and processing

Peptides were analyzed by LC-MS/MS using a Dionex UltiMate 3000 Rapid Separation nanoLC and a Q Exactive™ HF Hybrid Quadrupole-Orbitrap™ Mass Spectrometer (Thermo Fisher Scientific). Approximately 1 μg of peptide samples was loaded onto the trap column, which was 150 μm × 3 cm in-house packed with 3 μm C18 beads. The analytical column was a 75 μm × 10.5 cm PicoChip column packed with 3 μm C18 beads (New Objective, Inc.). The flow rate was kept at 300 nL/min. Solvent A was 0.1% FA in water and Solvent B was 0.1% FA in ACN. The peptide was separated on a 120-min analytical gradient from 5% ACN/0.1% FA to 40% ACN/0.1% FA. The mass spectrometer was operated in data-dependent mode. The source voltage was 2.10 kV and the capillary temperature was 320 °C. MS1 scans were acquired from 300–2000 m/z at 60,000 resolving power and automatic gain control (AGC) set to 3 × 106. The top 15 most abundant precursor ions in each MS1 scan were selected for fragmentation. Precursors were selected with an isolation width of 2 Da and fragmented by higher-energy collisional dissociation (HCD) at 30% normalized collision energy in the HCD cell. Previously selected ions were dynamically excluded from re-selection for 20 seconds. The MS2 AGC was set to 1 × 10^5^. All samples were run in duplicate.

### Proteomics validation via immuno-PCR (iPCR)

Protein was extracted from porcine ovary slices that were decellularized (as described above). Tissue was placed in a protein extraction buffer (1% SDS, 50 mM Ammonium Bicarbonate, 50 mM NaCl, Halt Protease Inhibitors (100 µL per 10 mL of buffer). Tissue was placed in reinforced 2 mL tubes (Omni International, 19–648) with 2.8 mm polycarbonate beads (Omni International, 19–646). Tissue was homogenized with an Omni BeadRuptor12 in a cold room (Omni International, 19-050 A) using the following settings: six cycles at speed 6.0, 45 s homogenization, 75 s delay between cycles. Homogenate subsequently sonicated on ice 3 times at 75% amplification for one minute. Samples were then centrifuged at 10k rpm for 15 minutes. A 1 mL aliquot was taken from the samples and sample was treated with an SDS-Out kit (Thermo Scientific, 20308). Protein concentration was measured using a BCA method (Fisher Scientific, PI23227). Samples were normalized to a concentration of 1,000 ng/µL using protein lysate buffer from the Taqman Open Kit (Thermo Fisher, 4453745).

Antibodies were biotinylated using EZ-Link Sulfo-NHS-LC-Biotin, No-Weight format kit (ThermoFisher,21327). Antibody information is listed in Suppl. Table 1. Excess biotin was removed using two cycles of filtration utilizing Zeba Micro Spin Desalting columns, 40k (Fisher Scientific, PI87765). Probes were then tested for suitability using method described under Thermo Fisher’s Taqman Protein Assays Probe Development Protocol (Thermo Fisher, 4448549). To compare protein quantity between depths a standard method described in Thermo Fisher’s Taqman Protein Assays Sample Prep and Assay (Thermo Fisher,4453745) was used. A four-point dilution series was used with 2000 ng of total protein then serially diluted 1:10. A qPCR machine (Applied Biosystems, QuantStudio 3) was used for running the assay. Because some samples had undetectable levels of protein, dose curve data are presented as change in CT over no template controls. Two-way ANOVA and multiple t-test based analysies were used.

### Gene expression analysis using real-time semi-quantitative PCR (qPCR)

Tissue was flash frozen in liquid nitrogen and stored at −80 °C prior to homogenization. Tissue underwent Proteinase-K digestion (3–4 hours, 37 C, Zymo, D3001-2-5) prior to homogenization using Beadruptor12 in a cold room (6 cycles at Speed 6.0, 45 s shake, 75 S delay between cycles). RNA extraction was done using Zymo mini-prep kit (Zymo, R1054). RNA preps were cleaned and concentrated using RNA-Clean up kit (Denville Scientific, Z5214). Total RNA (0.5 µg) was reverse transcribed into cDNA using Superscript IV Vilo kit (Life Technologies, 11756050). qPCR was carried out using a QS3 using standard SYBR reagent templates from manufacturer. Primer information is listed in Suppl. Table 2.

### Immunohistochemistry (IHC) analysis of matrisome protein candidates

Bisected porcine ovaries were fixed in Modified Davidson’s Fixative (MDF) at 4 °C and processed using an automated tissue processor (Leica) and embedded in paraffin. Deparaffinization was carried out using a soap-based method, as described previously^31^. Antigen retrieval was performed using citrate buffer (10 mM sodium citrate, 0.05% Tween 20, pH 6.0) in a pressure cooker for 35 minutes. Tissue was blocked using 2% donkey serum (Sigma-Aldrich, D9663), 1% BSA (Fisher Scientific, BP671), 0.1% cold fish skin gelatin (Fisher Scientific, NC9369923), 0.1% TritonX-100 (Sigma-Aldrich, P1379), 0.05% SodiumAzide (Fisher Scientific, AAJ62036K2), in PBS (Fisher Scientific, AAK62036K2). Tissue sections were incubated with primary antibody in block solution (2% Donkey Serum, 1% BSA, 0.1% Cold Fish Skin Gelatin, 0.1% Triton X-100, 0.05% Tween 20, 0.05% Sodium Azie, in 1X PBS; overnight, 1:200). Antibody information is listed in Suppl. Table 1. Staining visualized using 488 secondary antibody (1 hour incubation, 1:100, Novus Biological, NBP1-75292). Nuclear staining performed using propidium iodide (30 minutes, 1:1000, Sigma-Aldrich, P4864-10ML) or hoescht (30 minutes, 1:200, Sigma, B2261). Sudan Black incubation (15 minutes, 0.1%, Alfa Aesar, J62268-09) was used to reduce background. Stained sections were mounted using clear-mount with PIPES buffer (Electron Microscope, F6057-20ML). Stained sections were imaged (Keyence, BZ-X700), and analyzed using BZ-X Analyzer (Keyence). These analyses were performed across six experiments with at least two technical replicates each for three biological replicates (three ovaries from three different animals). At least two sections for each experiment were not incubated with the primary antibody and acted as our “no primary controls”. These sections were used to set the exposure settings for that fluorescent channel to reduce the background fluorescence and reveal secondary (fluorophore) binding to primary antibodies. Additional controls were used to confirm the specificity of each antibody to our protein of interest within the ovarian tissue section. Primary antibodies were incubated with 10 times more peptide (based on antibody molarity) and incubated at room temperature for 1 hour to block specific binding (Suppl. Table 2). The IHC protocol above was followed with these blocked antibodies (Suppl. Fig. 1).

### Proteomic data processing and statistical analysis

A total of 64 LC-MS/MS raw files were analyzed. Protein Tandem MS data was queried for protein identification and label-free quantification against the SwissProt *Sus scrofa* database using MaxQuant^32,33^. The following modifications were set as search parameters: peptide mass tolerance at 6 ppm, trypsin digestion cleavage after K or R (except when followed by P), two allowed missed cleavage site, carbamidomethylated cystein (static modification), and oxidized methionine, protein N-term acetylation (variable modification). Search results were validated with peptide and protein false discovery rate (FDR) both at 0.01. Proteomic stringency was increased by requiring: (1) all proteins to have more than one identified peptide, (2) proteins must have peptide reads in two planes to allow for intersections, (3) and peptides must have a read in each biological replicate to be considered for analysis. Proteins that passed these three rules were used for further analysis using a custom SAS script (see Supplemental Files). Peptide intensities were log transformed to control for multiplicative errors associated with FT-ICR MS. These intensities were then standardized to allow unbiased comparisons between peptides of the same protein. Least squares (LS) means were used to estimate differences in protein composition across depths, and differential expression in proteins. All annotation of data was performed utilizing Panther (http://www.pantherdb.org/, version 14.1, 2018) and MatrisomeDB (http://matrisomeproject.mit.edu/, version 2.0, 2016) databases, conversion of unknown IDs were performed using DAVID (https://david.ncifcrf.gov/, version 6.8, 2016). Statistical analyses for qPCR and iPCR data were performed using GraphPad Prism (version 8.1.2) software. qPCR results were subjected to unpaired, two-tailed t tests with α = 0.05 and iPCR data was subjected to a two-way ordinary ANOVA with α = 0.05 and multiple t tests where the statistical significance was determined by the Holm-Sidak method with α = 0.05; each group (cortex, medulla, etc.) was analyzed individually without assuming consistent standard deviations. All bar graphs display means with standard errors. Ranges of significance are described in each figure legend and P values for comparisons that are not significant are listed in the text.

### Literature search of matrisome proteins

Literature searches were performed using the National Institutes of Health (NIH) PubMed database (ncbi.nlm.nih.gov/pubmed) with the following search terms “((protein name) OR protein ID) AND ovary” and “((protein name) OR protein ID) AND ovarian”. Articles were scanned to determine if and how the protein was identified in the ovary and articles that investigated only non-mammalian species were omitted from further analysis. The remaining articles were reviewed to identify the function of the protein Suppl. Table 4. For those proteins that had no results that met the above criteria, the GeneCards human gene database (genecards.org) was searched for aliases to our protein names and IDs and additional searches were performed where necessary. Proteins without identified articles that include characterization or potential function of that protein within the mammalian ovary have “none” and “unknown” in the corresponding fields. Proteomic analysis data on ovarian tissue or cells that were provided as figures or supplemental spreadsheets were searched using protein names and protein IDs.

## Results

### Ovarian processing

We sought to identify and spatially map the matrisome of porcine ovaries. Bisected porcine ovaries were processed sagittally and axially into uniform 0.5 mm slices (to 3.5 mm depth) to create intersecting planes that together created complete coverage of 1/8^th^ of the organ (Fig. 1a,b). The cortical region that contains the primordial follicle pool is within the first 0.5 mm slice (Suppl. Fig. 2). Samples were decellularized prior to LC-MS/MS and iPCR analyses and native samples were used for comparative mRNA expression (Fig. 1a). A total of 6,711 peptides were detected with an FDR < 0.01, these peptides were identified as belonging to 615 proteins. Following data filtration with our three stringency rules, the remaining 440 proteins were analyzed using our SAS-based bioinformatics pipeline (see Suppl. SAS script). 134 proteins were significantly differentially expressed across depths with LS-means.

### Classification of identified proteins

**Figure 2:**
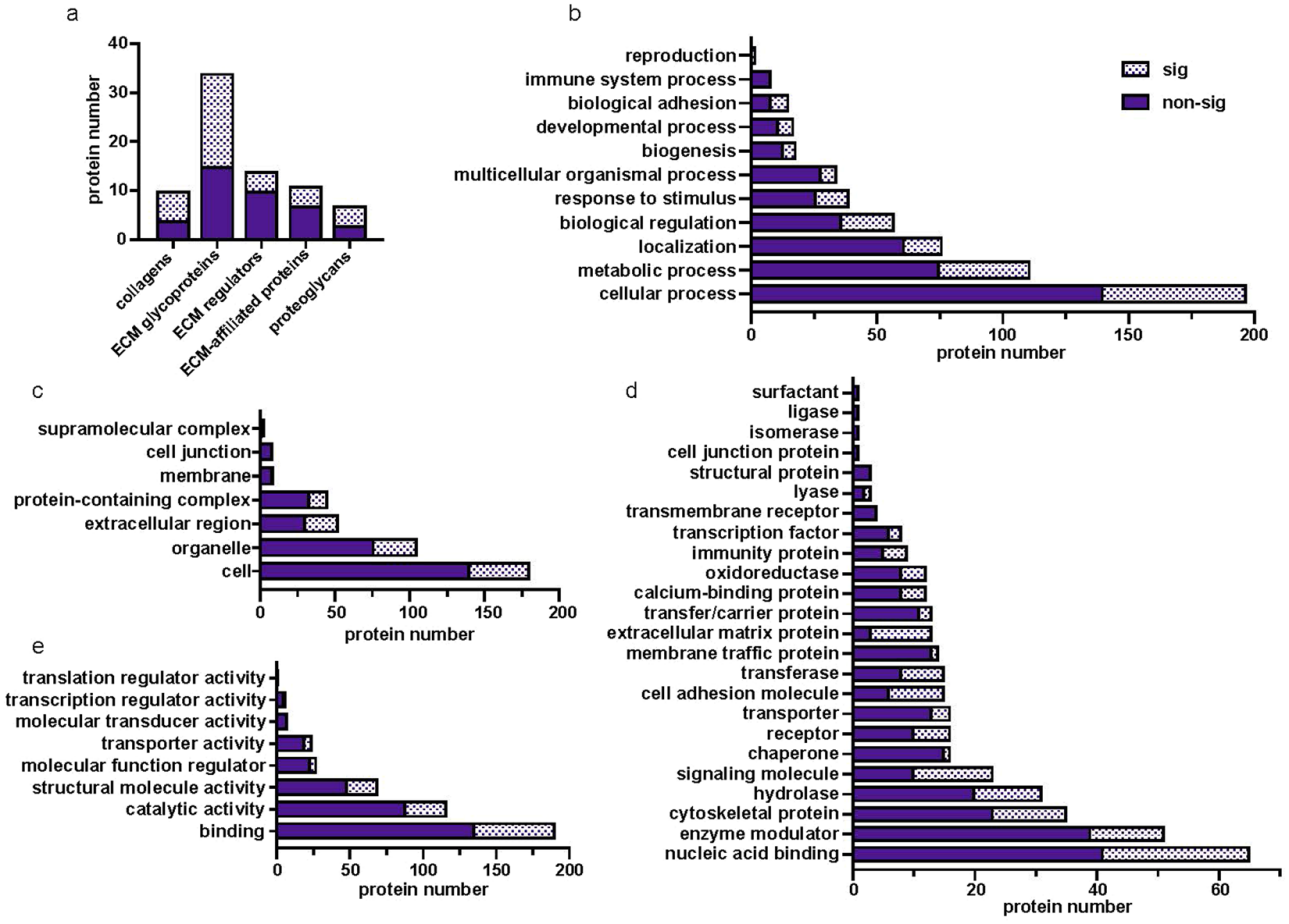
Number of proteins identified within (**a**) matrisome (**b**) biological process, (**c**) cellular component, (**d**) protein class, and (**e**) molecular function categories. sig, significantly differentially expressed.

Of the 440 proteins identified, 82 are core matrisome (collagens, proteoglycans, ECM glycoproteins) and ECM-associated (ECM regulators, secreted factors, and ECM-affiliated proteins) proteins (Fig. 2a). Secreted factors, including a calcium binding protein (S100A11) and the growth factor pleiotrophin (PTN), were identified in our analysis; however, they failed to pass the stringency filters. Of the 82 matrisome proteins 42 were shown to be significantly differentially expressed across depths. The core matrisome included 55 and 32 proteins for our total and significantly differentially expressed proteins respectively. Our data includes 11 collagens, 7 of which were differentially expressed; 36 ECM glycoproteins, 20 of which were differentially expressed, and 8 proteoglycans, 5 of which were differentially expressed. The matrisome-associated proteins included 16 ECM regulators and 11 ECM-associated proteins of which 6 ECM regulators and 4 ECM-affiliated proteins were significantly differentially expressed.

Gene ontology analysis subdivided the proteins into 11 biological process (Fig. 2b), 7 cellular component (Fig. 2c), 24 protein class (Fig. 2d) and 8 molecular function (Fig. 2e) categories. The proteins represented localization, response to stimulus and reproduction processes (Fig. 2b). Specifically, zona pellucida proteins were identified, which are specialized ECM glycoproteins that surround the oocyte. Proteins within both the intracellular and extracellular compartments were identified including nucleic acid binding proteins, organelle, extracellular matrix, extracellular region, and cell junction proteins (Fig. 2c,d). Most proteins that were identified as signficantly differentially expressed were within the nucleic acid binding protein class (17.2%), followed by extracellular matrix proteins (3.4%), cytoskeletal proteins (9.3%), cell junction (0.3%), cell adhesion (4.0%), and structural proteins (0.8%, Fig. 2d). 80% of all gene ontology categories contained at least one protein that was significantly differentially expressed across depths (Fig. 2b–e).

### Validation of select proteins

A selection of core ECM proteins were further evaluated and validated. We chose collagens I (COL1A2) and IV (COL4A2), Agrin (AGRN), Extracellular Matrix Protein 1 (ECM1), Elastin Microfibril Interfacer 1 (EMILIN1), Fibronectin (FN1), Transforming Growth Factor Beta 1 (TGFB1), Vitronectin (VTN), and Zona Pellucida Glycoprotein 3 (ZP3). LS means data from both sagittal and axial slices were combined to determine standard deviations from LS means for each protein across depths (0.5 mm – 3.5 mm, Suppl. Fig. 3). COL1 and EMILIN1 expression was consistently lower in 1.0–3.5 mm slices when compared to 0.5 mm expression. COL4A2 was consistent across depths. AGRN, FN1, TGFB and ZP3 had a peak in expression around 1.5 mm, while VTN had a peak at 2.0 mm. ECM1 had undetectable levels in the 0.5 mm slices and remained relatively consistent at 1.0–3.0 mm.

**Figure 3:**
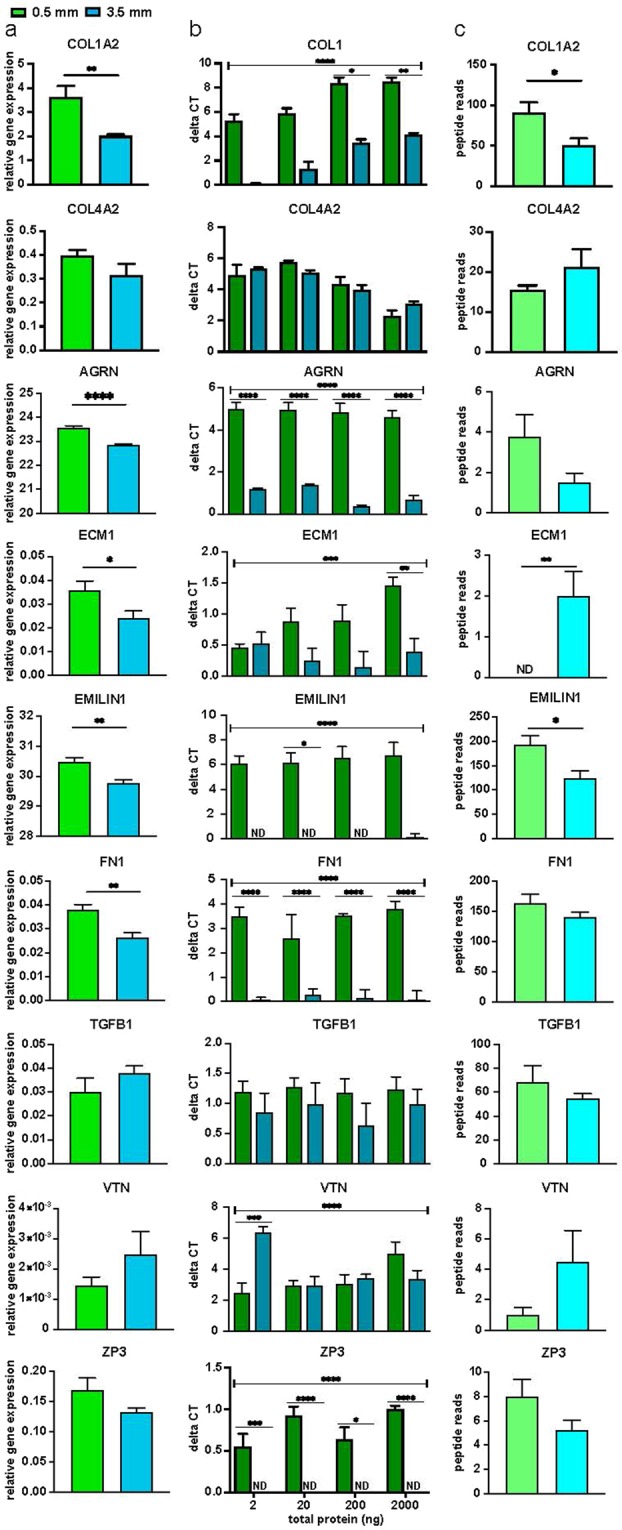
Comparison of protein or gene expression between most superficial and deepest slice. A selection of matrisome proteins were evaluated within 0.5 mm and 3.5 mm slices using (**a**) qPCR (n = 4–6 per slice), (**b**) iPCR (n = 4), and (**c**) peptide reads (n = 8 per slice, 4 ovaries with 2 technical replicates each). Bars represented as mean, SEM; *P < 0.05; **P < 0.005; ***P < 0.0005; ****P < 0.0001; ND, not detected.

The results of these selected proteins were validated by comparing the most superficial (0.5 mm, cortex) to the deepest slices (3.5 mm, containing deep medulla) using qPCR and iPCR and relating these to peptide reads from the proteomic analysis at the same depths (Fig. 3). While all proteins chosen for this analysis were significantly differentially expressed across depths (0.5 mm–3.5 mm), except for COL4A2, a comparison of the 0.5 mm to 3.5 mm samples did not always produce significant differences. AGRN (p = 0.083), and ZP3 (p = 0.110) were trending towards greater peptide reads in 0.5 mm samples compared to 3.5 mm, while COL1A2 and EMILIN1 were significantly more highly expressed in the 0.5 mm samples (Fig. 3c). ECM1 (p = 0.005) and VTN (p = 0.140) were more highly expressed in 3.5 mm samples, and COL4A2 (p = 0.228), FN1 (p = 0.216) and TGFB1 (p = 0.368) were not significantly differentially expressed by our analysis (Fig. 3c). Transcriptional and iPCR analyses that matched the statistical significance of the peptide reads were considered similar to the proteomics data. From the pool of selected proteins, 6/9 proteins, COL1A2, COL4A2, EMILIN1, AGRN, VTN, and ZP3, had similar gene expression (Fig. 3a) and iPCR profiles (Fig. 3b) to the proteomics results (Fig. 3c). One protein, FN1 was expressed significantly more in the 0.5 mm slices by qPCR and iPCR, but this difference did not reach significance when comparing the peptide reads from these slices. Additionally, 2/9 proteins, TGFB1 and ECM1, did not match with expected trends based on our peptide reads. ECM1 had significantly higher protein abundance and higher gene expression at 0.5 mm and 3.5 mm counter to the expected trend based on the peptide reads.

### Spatial composition of ovarian matrisome

**Figure 4:**
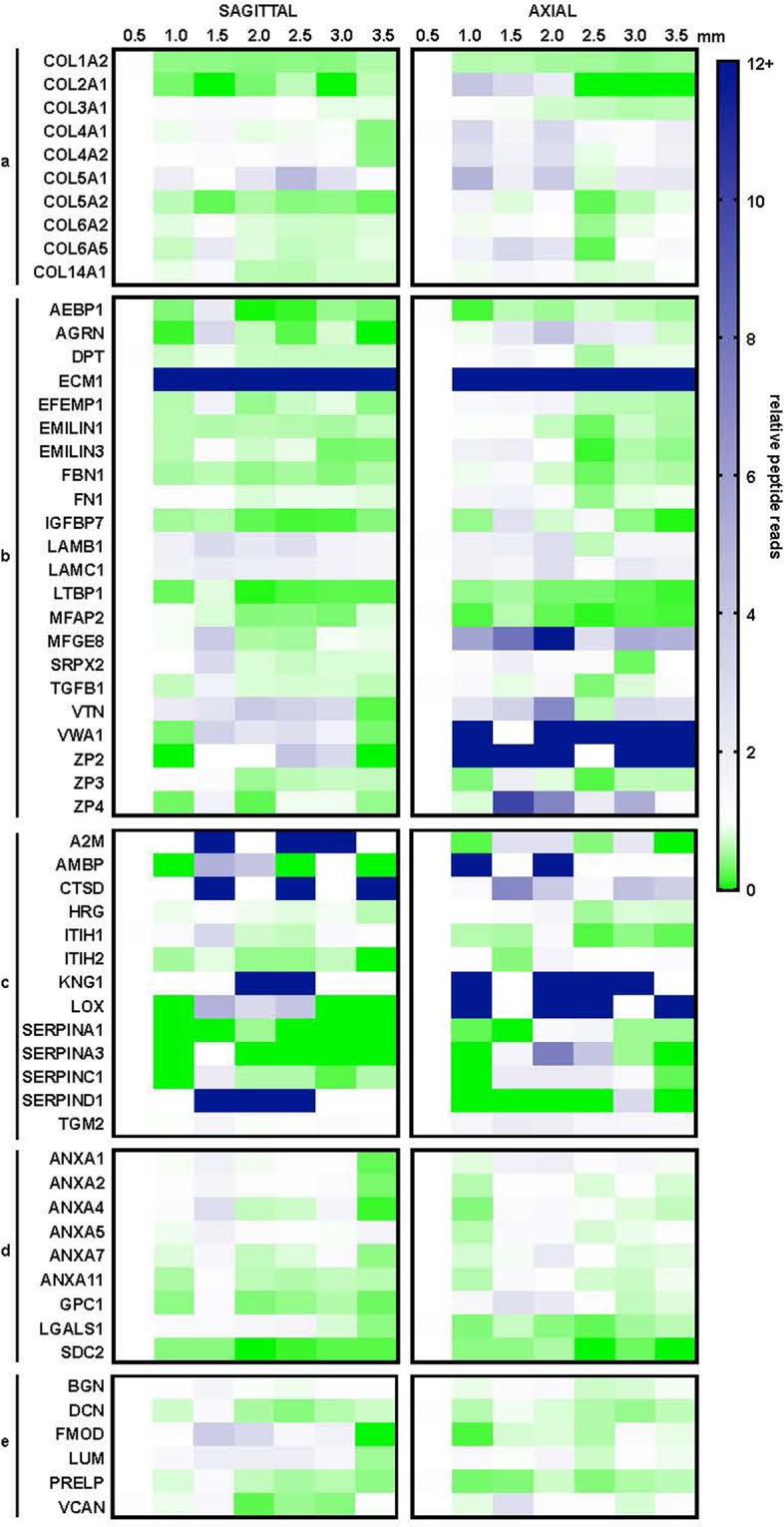
Relative protein reads of matrisome proteins. Proteins within matrisome categories, (**a**) collagens, (**b**) ECM glycoproteins, (**c**) ECM regulators, (**d**) ECM-affiliated proteins, (**e**) proteoglycans are represented as relative reads to the average reads at 0.5 mm.

Proteins were grouped into their matrisome protein categories and examined across depths in the two anatomical directions (Fig. 4). In our data, there was greater expression of ECM-affiliated proteins (Fig. 4d) and proteoglycans (Fig. 4e) in the cortex (0.5 mm) than the majority-medulla slices (1.0 mm–3.5 mm). Some proteins, such as COL1A2, EMILIN1 and SDC2 had consistently less protein reads across 1.0–3.5 mm depths in comparison to 0.5 mm (cortex slice) in both sagittal and axial processing methods. Other proteins, such as COL5A1, ECM1 and LAMC1 had consistently more protein reads in the majority medulla (1.0–3.5 mm) samples than the cortex (0.5 mm). Still other proteins were more heterogeneous or did not produce consistent trends in expression across ovarian depths or between sagittal and axial processing.

### Localization of Protein candidates

**Figure 5:**
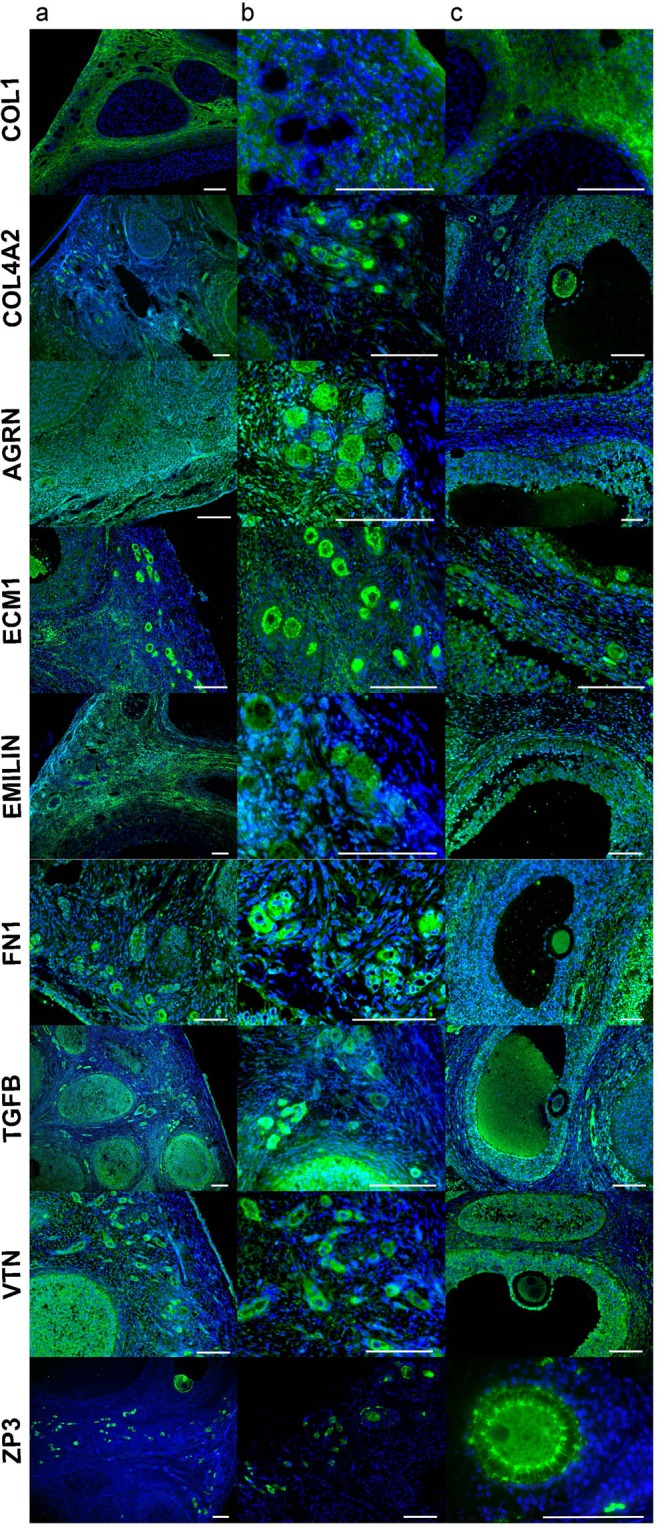
IHC analysis of selected proteins in (**a**) an overview across compartments, (**b**) small follicles, and (**c**) growing follicles. Scale bar, 100 µm.

We identified the protein distribution of nine proteins across ovarian compartments and within ovarian cells with IHC. Qualitative examination of these proteins as an overview across compartments (Fig. 5a) and within small (Fig. 5b) and growing follicles (Fig. 5c), revealed that COL1, AGRN, FN1, TGFB and VTN are abundantly expressed in the ovarian surface epithelium (OSE). Most proteins were localized to at least some stromal cells; ZP3 did not. ECM1 and FN1 appeared more abundant in medullary over cortical stromal cells. COL4A2 was localized to granulosa cells of small and growing follicles and antral follicle oocytes. EMILIN1, FN1, TGFB and VTN were also localized to granulosa cells of small and growing follicles. AGRN was expressed in all cell types including oocytes and granulosa cells, while ECM1 expression was localized to the outer perimeter of oocytes. FN1 was localized to the outer perimeter of small oocytes. ZP3 was expressed in the oocyte and was more highly localized to the transzonal projections in large growing follicles.

### Protein expression across compartments

**Figure 6:**
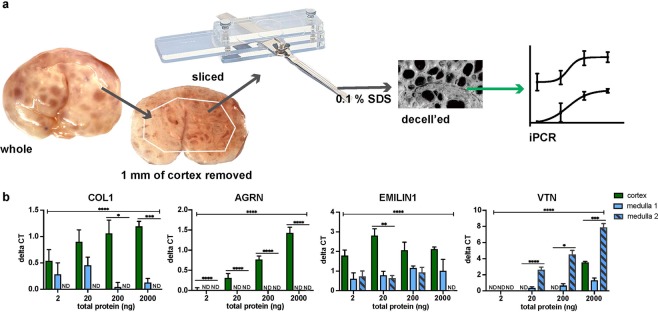
Schematic of (**a**) processing the porcine ovary by removing ~ 1 mm from the ovarian surface followed by a tissue slicer, prior to decellularization then iPCR analysis. (**b**) COL1, AGRN, EMILIN1 and VTN protein expression in cortex region and slices of medullary tissue (n = 4 per slice). Bars represented as mean, SEM; *P < 0.05; **P < 0.005; ***P < 0.0005; ****P < 0.0001. iPCR, immuno PCR; ND, not detected.

We used an additional processing technique, method 2, to examine protein expression differences across the defined ovarian compartments. We removed the first 1 mm of tissue using a Stadie-Riggs slicer, and then trimmed 1 mm of tissue from the edge of the remaining tissue. From the remaining tissue, we obtained two slices in the sagittal orientation, which we refer to as intermediate (medulla 1) and deep medulla (medulla 2, Fig. 6a). This produces a lower resolution (1 mm and fewer slices) in comparison to processing method 1 but removes the cortical region from the medullary slices (represented as 1.0 mm–3.5 mm in method 1).

We chose four proteins to investigate, COL1, AGRN, EMILIN1, and VTN (Fig. 6b) by iPCR. All proteins had similar expression patterns to the previous methods with COL1, AGRN and EMILIN1 having higher abundance in the cortex compared to the medulla and VTN had higher abundance at the medullary slices compared to the cortex. This indicates that the small amount of cortex present in medullary slices used in our proteomic analysis does not greatly alter the protein composition signature of the compartment.

### Visualization of COL1A2 and EMILIN1 expression across intersecting planes

**Figure 7:**
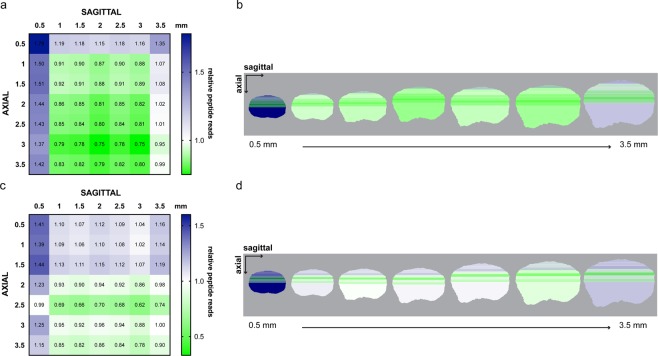
Heatmaps of (**a,b**) COL1A2 and (**c,d**) EMILIN1 expression at sagittal and axial intersections (**a**,**c**) relative to univeral mean of peptide reads. (**b,d**) Schematic representation of expression at axial depth slices (top to bottom) visualized on each sagittal slice (left to right).

To visually represent the relative expression of COL1A2 and EMILIN1 spatially across the ovarian area analyzed, we normalized the peptide reads for each protein to its universal mean (Fig. 7a,c). This was then represented visually as axial slices superimposed over each sagittal slice from 0.5–3.5 mm (Fig. 7b,d). Because the samples analyzed only represent half of the ovary in each direction, the axial slices covered half of each sagittal slice. There were patterns of increased expression in the cortical regions of both protein maps represented by the 0.5 mm sagittal column and 0.5 mm axial row. This was more confined to the 0.5 mm depth in both directions in COL1A2 in comparison to EMILIN1, which had an increase in expression to the 1.5 mm depth in the sagittal direction.

## Discussion

This work describes a pipeline for mapping matrisome proteins across an ovary and could be utilized in other organs to identify spatial or compartmental differences in matrisome protein content. With this analysis, the quantity of proteins can be represented at a resolution of 0.5 mm × 0.5 mm × ovary width with full coverage of 1/8^th^ of the ovary. The processing techniques utilized previously described decellularization methods that had removed significant cellular contents while maintaining the integrity of the scaffold as detected by scanning electron microscopy^7^. This method enriches matrisome proteins and reveals their relative spatial quantities^34^. While the decellularization and stringent criteria used for our analyses are meant to reveal robust matrisome proteins within the ovary, these techniques may omit important proteins. Additionally, modifications that would reveal the activity status of a protein are also not revealed in bottom-up proteomics analyses. However, these data have provided novel insights into matrisome quantity and compartmentalization and experiments that reveal the roles of matrisome proteins in controlling folliculogenesis were selected from this analysis and are ongoing.

While pigs are poly-ovulatory mammals, and humans are mono-ovulatory, there were several reasons for choosing porcine ovaries for this study. Unfortunately, there are no perfect models for human ovaries; some monkeys have premeiotic germ cells after birth and monkey ovaries are smaller than human ovaries and would not result in the same resolution that we achieve with our 0.5 mm slicer of a larger ovary^35–37^. The follicular waves are more similar in porcine ovaries to humans than other mono-ovulatory species like cows or sheep, which have short follicular, versus luteal phases and more intraovulatory waves than humans^35^. These may be important distinctions as we interrogate the effects of these matrisome proteins on folliculogenesis. There may be universal and intra-compartmental differences in matrisome quantity and composition as the ovary grows in size and develops from prepubertal ovaries that contain primordial follicles exclusively to postpubertal ovulating ovaries. The ovaries that were analyzed were from peripubertal animals, where they are not reproductively mature and are not cycling, but there were follicles present at each stage within the tissue (Suppl. Fig. 2). The primordial follicle compartment was also within the first slice of 0.5 mm and was important for this analysis. Finally, there is significant precedent for using porcine materials in human regenerative medicine^38–41^, which is an important consideration for our planned downstream applications of utilizing this information to create informed biomaterial inks for an engineered ovarian transplant.

Ovary processing method 1, where the organ is sliced in a uniform manner, resulted in the 1.0–3.5 mm slices containing some cortical tissue (at 0–0.5 mm from the edge). Because the differences in the cortical versus the medullary compartment may be essential in identifying proteins that influence quiescence versus activation and growth of ovarian follicles, we chose to test an additional processing method. This method 2 would produce a map at a lower resolution but removes the cortical tissue from the analysis of medullary slices. We found that COL1, AGRN, EMILIN1 and VTN protein expression was consistent with the comparison of the 0.5 mm to 3.5 mm slices from the previous method, indicating that the cortical tissue did not significantly contribute to the analysis of the mostly-medulla slices. Additionally, some differences were detected between the intermediate (medulla 1) and deep medulla (medulla 2), indicating that the heterogeneous expression of proteins within the medulla, demonstrated by the sagittal and axial heatmaps, is preserved during this processing method and is not solely attributed to the addition of cortical tissue in these slices.

There have been previous reports examining the ovarian proteome with one focusing on the ovarian matrisome^24,25^. However, this study focused solely on the human ovarian cortex and did not examine the composition of the medulla. Our study and the previous study performed on cortical tissue from human ovaries, both found a similar number of matrisome proteins (82 versus 85, respectively) and they were dispersed among the same matrisome sub-categories^25^. The previous study found four secreted factors, including S100 proteins; however, these proteins were not detected or were removed here for failure to meet the stringent analytical criteria. This exclusion may be due to the reduction in peptide reads due to the decellularization treatment of our ovarian slices prior to proteomic analysis. The proteomic analysis described here identified matrisome proteins that were previously not identified in mammalian ovaries or have only been identified in transcript screens including collagen subunits (COL5A1, COL5A2, COL6A5), ECM glycoproteins (AEBP1, AGRN, DPT, EMILIN3), ECM-affiliated proteins (ANXA7, GPC1, SDC2) and a proteoglycan (BGN) (Suppl. Table 4). Additionally, this study aimed to identify differences in matrisome protein abundance between ovarian compartments and was able to spatially map these across depths of the ovary.

The spatial analysis performed here revealed proteins that may control folliculogenesis. The largest number of matrisome proteins that were identified and were significantly differentially expressed across depths were glycoproteins, a class of matrisome proteins that can facilitate the translation of the extracellular environment into signal transduction cues^22^. Our dataset revealed two glycoproteins, AGRN and EMILIN1, which have not been functionally described in the normal ovary, but have been shown to be involved in important pathways that translate extracellular or mechanical signals into cellular responses via Hippo and PI3K-AKT associated pathways in other organs^42,43^. Both of these proteins were significantly differentially expressed across depths and compartments in the ovary. However, EMILIN1 expression decreased deeper into the ovary, while AGRN (FN1 and TGFB) had peaks in protein reads at 1.5 mm depths (Suppl. Fig. 3c,g). This peak may correspond to an area with more growing follicles, as each of the proteins were localized to follicles by IHC and this pattern matches with that of ZP3^44^ (Suppl. Fig. 3i). Localization of the protein to follicular cells versus stromal cells will be important to identifying structural support proteins that may be used for creating biomaterial scaffolds for isolated follicles. FN1 expression appears greater in the cortical slices, by qPCR and iPCR of 0.5 versus 3.5 mm slices (though not significantly different by peptide reads for these slices). However, FN1 may be more useful as a structural protein for creating the medulla, as it is localized by IHC to stroma around growing follicles^7,45^.

In addition to biochemical signals and structural support, matrisome proteins also provide physical cues. Compression of primordial follicles is required for them to remain quiescent in murine ovaries^46^. Digestion of the ovary with collagenase IV and trypsin released the primordial follicles from the ECM-associated stress as indicated by loosening of actin stress fibers and a progression in granulosa cell morphology from squamous to cuboidal. Growing these enzyme-treated ovaries in a pressure chamber reversed this phenotype and the primordial follicles were maintained in the cortex of the ovary^46^. Future studies linking how the matrisome composition directly relates to the physical forces on the follicles and the resulting follicle behavior will reveal essential protein composition, ratios and localization in relation to quiescent versus growing follicles. The technique described here looks to provides a unique toolset to identify matrisome proteins that are differentially expressed depending on compartment and spatial location. This pipeline will play a key role in identifying essential proteins that influence folliculogenesis to better inform biomedical engineering approaches to tissue regeneration.

## Supplementary information

Supplemental Figures and Tables

Supplemental Dataset 1

Supplemental Dataset 2
