# Supplementary material for: Proteomic analyses of decellularized porcine ovaries identified new matrisome proteins and spatial differences across and within ovarian compartments
Source: Sci Rep. 2019 Dec 27;9:20001. doi: 10.1038/s41598-019-56454-3 (PMC6934504; doi:10.1038/s41598-019-56454-3)
Supplement: Supplementary file 1 — Supplemental Figures and Tables [file 41598_2019_56454_MOESM1_ESM.pdf]

Proteomic analyses of decellularized porcine ovaries identified new matrisome proteins and spatial differences across and within ovarian compartments.

Nathaniel F. Henning[1,2], Richard D. LeDuc[3], Kelly A. Even[1,2], Monica M. Laronda[1,2]

Affiliations: [1]Department of Pediatrics, Feinberg School of Medicine, Northwestern University; [2]Stanley Manne Children's Research Institute, Ann & Robert H. Lurie Children's Hospital of Chicago; [3]Proteomics Center of Excellence, Northwestern University

Supplemental Figure 1

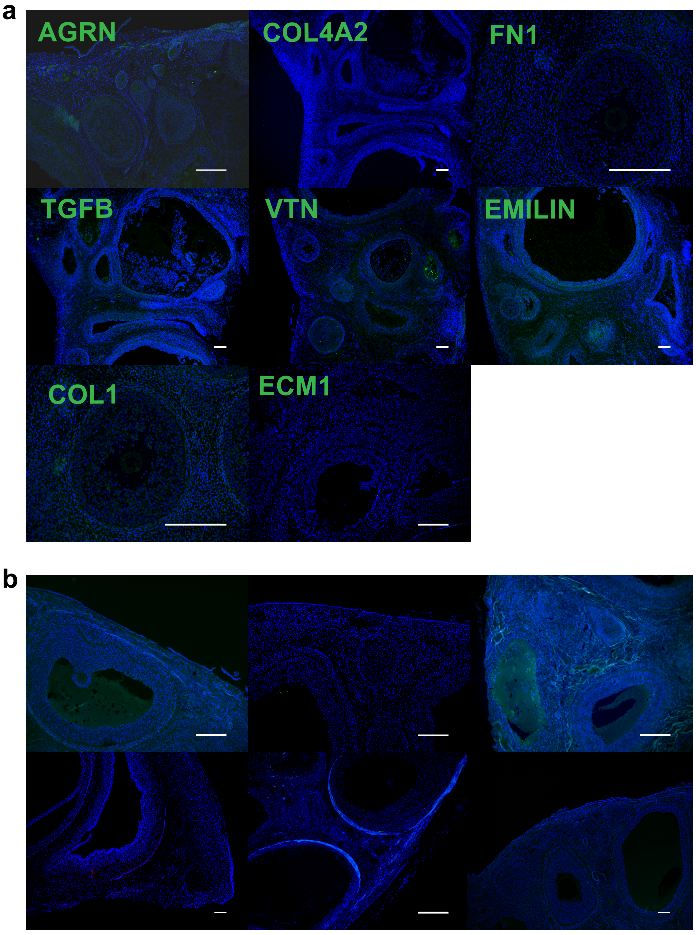

IHC controls were used in our study. (a) Peptides for the protein of interest (green) were used to reveal off-target binding within the porcine ovarian tissue and (b) no primary controls were used for each experiment to reveal non-specific binding of secondary antibodies (green). All were counterstained with a nuclear stain (blue). Scale bar, 200 µm.

Supplemental Figure 2

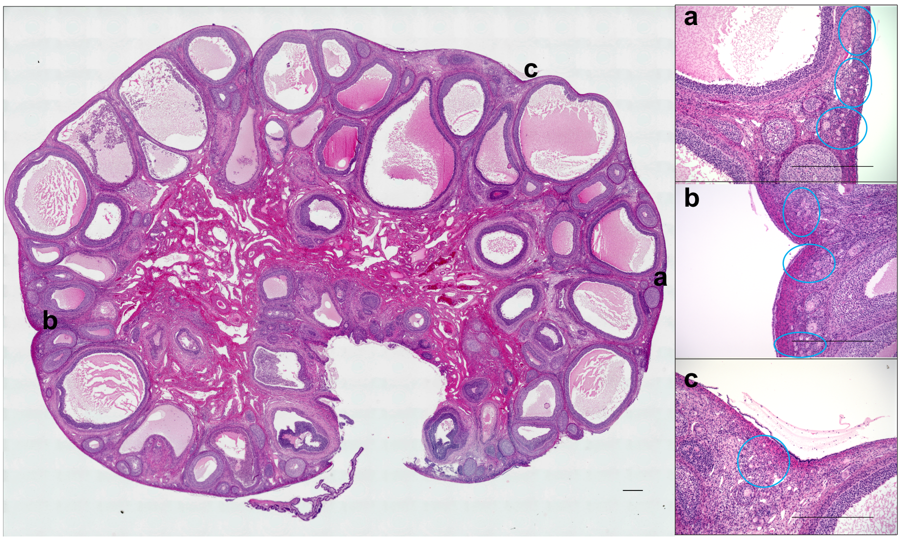

Hematoxylin and eosin stained section of a peri-pubertal porcine ovary demonstrating follicles at all stages of maturation, but lacking corpus lutea. Inset images were taken from locations within the whole ovary scan with the matching letter. Clusters of primordial follicles (blue ovals) were present within 0.5 mm from ovarian surface. Scale bar, 0.5 mm. Note: the ovaries shrunk to ~ 50 - 60% of the original size through fixation, processing and embedding.

Supplemental Figure 3

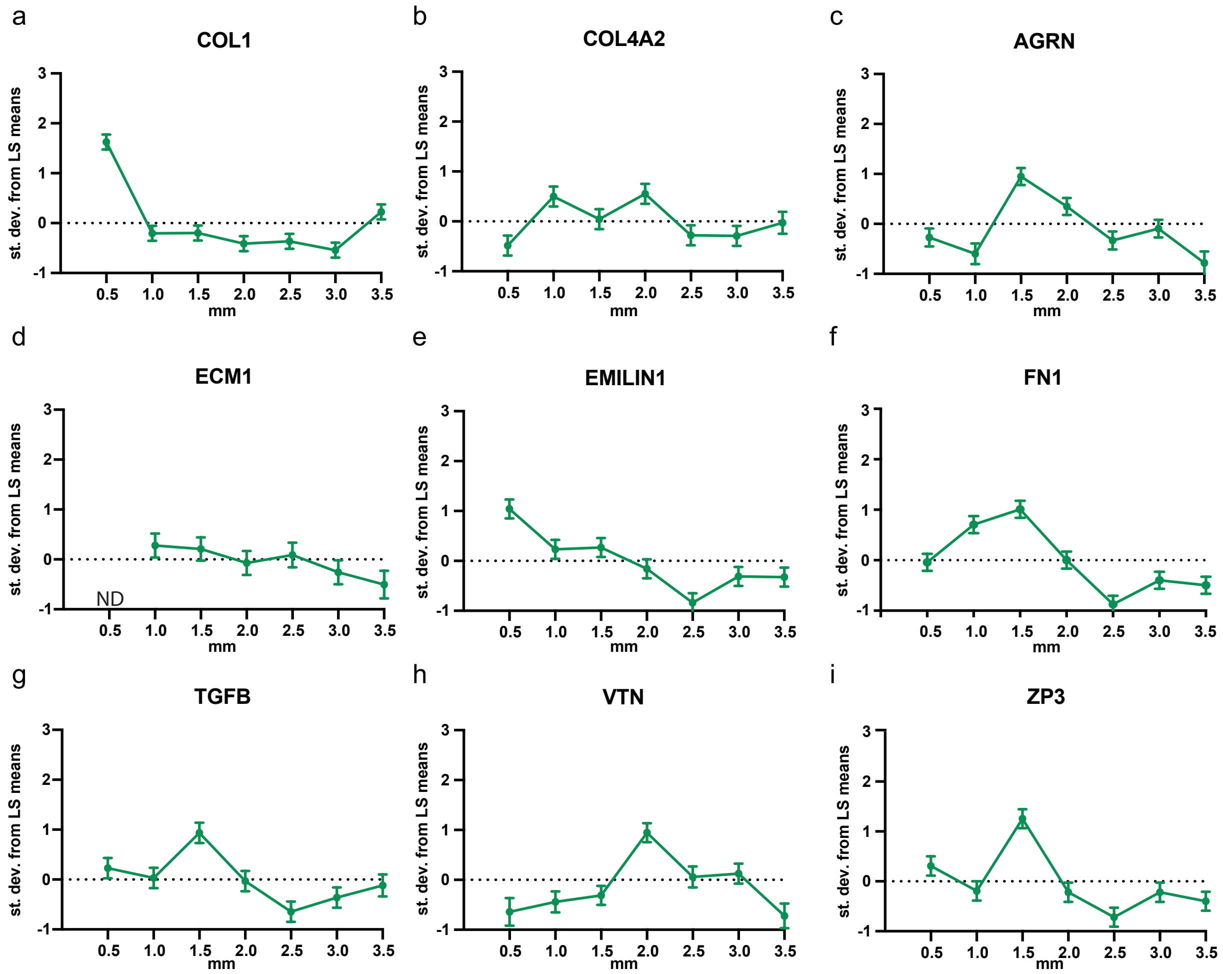

Peptide reads represented by standard deviations from LS Means for (a) COL1, (b) COL4A2, (c) AGRN, (d) ECM1, (e) EMILIN1, (f) FN1, (g) TGFB, (h) VTN, (i) ZP3. These are represented across depths 0.5 - 3.5 mm (n = 8 per slice, 4 ovaries with 2 technical replicates each); ND, not detected; bars represented as mean, SEM.

Supplemental Table 1: Antibodies used for iPCR and IHC

Target	Manufacturer	SKU	Use
AGRN	Abcam	ab85174	iPCR
AGRN	Lifespan Biosciences	LS-G81348-20	IHC
COL1	Abcam	ab90395	iPCR
COL1	EMD Millipore	234138-1MG	IHC
COL4	EMD MIllipore	CC083	IHC
COL4A2	Abclonal	A7657	iPCR
ECM1	Abcam	ab234976	iPCR & IHC
EMILIN1	Fisher Scientific	PA551745	iPCR
EMILIN1	ABNova	H00011117P01	IHC
FN1	Abcam	ab23750	iPCR & IHC
LMNA	Proteintech	10298-1-AP	iPCR & IHC
TGFB	Abcam	ab92486	iPCR & IHC
VTN	Abcam	ab140016	iPCR & IHC
ZP3	Fisher Scientific	50-561-353	iPCR & IHC

Supplemental Table 2: Primers used for qPCR

Target	Manufacturer	SKU
AGRN	Bio-Rad	qBtaCID0012730
COL1A1	Bio-Rad	qSscCED10042976
COL1A2	Bio-Rad	qSscCED0020342
EMILIN1	Bio-Rad	qSscCED0008548
COL4	Bio-Rad	qSscCID0013145
FN1	Bio-Rad	qSscCID0003939
ECM1	Bio-Rad	qSscCID0013839
VTN	Bio-Rad	qSscCED0021774
ZP3	Bio-Rad	qSscCID0013229
TGFB	Bio-Rad	qSscCID0018090
LMNA	Bio-Rad	qSscCID0012088

Supplemental Table 3: Blocking peptides/proteins used for IHC controls

Target	Manufacturer	SKU
AGRN	LifeSpan Biosciences	LS-G81348-20
COL1	EMD Millipore	234138-1MG
COL4	EMD Millipore	CC083
ECM1	R&D Systems	3937-EC-050
EMILIN1	Abnova	H00011117P01
FN1	EMD Millipore	341631-1MG
TGFB	R&D Systems	7754-BH-005
VTN	STEMCELL Technologies	7180

Supplemental Table 4: Matrisome proteins identified in the ovary

Protein ID	Previously identified in ovary	Characterized in ovary	Role in ovary
**COL1A2**	[1] (h)	[2–4] (m), [5] (h), [6] (b)	major structural component of organ
**COL2A1**	*	[7](r)	in theca and GC
**COL3A1**	[8] (b)	[5] (h)	inner layers of capsular stroma
**COL4A1**	[1] (h), [9,10] (b)	[5] (h), [3,4] (m), [6] (b)	basal lamina component
**COL4A2**	[1] (h), [9,10] (b)	[5] (h), [3,4] (m), [6] (b)	basal lamina component
**COL5A1**	[11] (h culture, *mRNA*)	none	unknown
**COL5A2**	[12] (b GC and theca, *mRNA*)	none	unknown
**COL6A2**	[1,13] (h), [14] (mo), [15] (m)	[16] (h, *undefined subunit of* $\alpha 6$),	localized to theca
**COL6A5**	*	[16] (h, *undefined subunit of* $\alpha 6$)	localized to theca
**COL14A1**	[1] (h), [14] (mo)	none	unknown
**AEBP1**	none	none	unknown
**AGRN**	none	none	unknown
**DPT**	[17](h, *"faint" mRNA*)	none	unknown
**ECM1**	[18] (p), [19] (h)	[19] (h)	downregulated in insulin resistant PCOS, potential antral arrest
**EFEMP1**	[18] (p)	[20] (h)	increased tumor angiogenesis, tumor progression
**EMILIN1**	[1,13] (h)	none	unknown
**EMILIN3**	none	none	unknown
**FBN1**	[1] (h), [8] (b)	[21] (fetal b, h), [22] (p), [23,24] (b), [24] (h)	TGFB regulation, structural component of elastin fibers and microfibrils, CC apoptosis
**FN1**	[1] (h), [18] (p)	[3,4,25] (m), [6] (b)	luteinization and CC expansion
**IGFBP7**	*	[26,27] (r)	steroidogenesis
**LAMB1**	[1] (h)	[28] (p), [19] (h), [3,4] (m)	cell proliferation, migration, downregulated in PCOS,
**LAMC1**	[1] (h)	[28] (p), [19] (h), [3,4] (m)	associated with premature ovarian failure, cell proliferation, migration
**LTBP1**	*	[29,30] (m), [23] (b)	modulation of TGFB1
**MFAP2**	[1] (h)	none	unknown
**MFGE8**	*	[31–33] (m)	phagocytosis of apoptotic GC, gonadogenesis
**SRPX2**	[18] (p)	none	unknown
**TGFB1**	[1] (h), [18] (p), [8] (b)	[34] (r), [35] (m), [36] (p)	cell growth, proliferation, inflammation, differentiation, apoptosis
**VTN**	[1] (h), [18] (p)	[37,38](h), [39,40] (h cells), [41] (p),	IGF binding, integrin binding and adhesion, cancer progression and metastasis
**VWA1**	*	[42] (m), [43–47](h)	interacts with ERK5-PI3K/Akt axis, increased expression in PCOS, cancer, and during pregnancy, stimulates platelet aggregation
**ZP2**	*	[48] (h, CHO), [49–52] (h), [53] (c), [54] (mo), [55] (m, mo,h), [56,57] (m)	oocyte maturation, fertilization
**ZP3**	*	[48] (h, CHO), [49–52] (h), [53] (c), [54] (mo), [55] (m, mo,h), [56,57] (m)	oocyte maturation, fertilization
**ZP4**	[14] (mo)	[48] (h, CHO), [49–52] (h), [53] (c), [54] (mo), [55] (m, mo,h), [56,57] (m)	oocyte maturation, fertilization
**A2M**	[1] (h), [18] (p)	[58–60] (h serum), [61] (r)	expressed in GC, increased in women with inflammatory conditions including neoplastic lesions, reduced expression in ovarian cancer
**AMBP**	[18] (p)	[62] (h)	interacts with ITIH family genes in solid tumor cancers
**CTSD**	*	[63,64] (h)	lysosome activation in late corpus luteum, oxidative stress in ovarian cancer
**HRG**	[14] (mo)	[65] (h), [66] (p), [67] (h follicle, embryo) [68] (h cells)	*in vitro* maturation, cancer cell invasion
**ITIH1**	[1] (h), [18] (p), [69] (b FF)	[62] (h)	solid tumor cancer progression, covalent linkage to hyaluronan for ECM stability
**ITIH2**	[1] (h), [18] (p), [69] (b FF)	[62] (h)	solid tumor cancer progression, covalent linkage to hyaluronan for ECM stability
**KNG1**	*	[70] (b), [71] (h serum, cells)	ovulation, stimulated by progesterone
**LOX**	*	[72] (p GC), [73] (h FF, h GC, r), [74] (h FF), [75] (h GC), [76–78] (m), [77] (b), [79] (r)	follicle development, angiogenesis in GC, TGFB, estrogenesis
**SERPINA1**	[1] (h), [18] (p)	[80] (m), [81] (h FF), [82] (h GC)	plasminogen activator inhibitor, upregulated in PCOS reducing plasmin levels
**SERPINA3**	[1] (h)	[80] (m)	plasminogen activator inhibitor
**SERPINC1**	[1] (h), [18] (p)	[80] (m), [83] (r serum)	plasminogen activator inhibitor
**SERPIND1**	[1] (h), [18] (p)	[80] (m)	plasminogen activator inhibitor
**TGM2**	[14] (mo), [84] (b, *mRNA*)	none	unknown

Protein ID	Previously identified in ovary	Characterized in ovary	Role in ovary
**ANXA1**	[1,13] (h), [14] (mo), [18] (p)	none	implicated in both cancer and PCOS, target of GNRH in gonadotrope cells, CL regression
**ANXA2**	[1,13] (h), [14] (mo)	none	unknown
**ANXA4**	[1,13] (h), [14] (mo)	none	unknown
**ANXA5**	[1,13] (h), [14] (mo), [85] (h blood)	[86] (r)	in GC after hCG
**ANXA7**	none	none	unknown
**ANXA11**	[13] (h)	none	unknown
**GPC1**	none	none	unknown
**LGALS1**	[14] (m)	[87] (b),[88,89] (m), [90] (h cells), [91] (b), [92] (p gc)	lutealization, regression of CL
**SDC2**	[93] (o CC, *mRNA*)	[94] (h)	confined to stroma of normal and benign tissue
**BGN**	[15] (m, *mRNA*)	none	unknown
**DCN**	[1] (h); [14] (mo), [18] (p), [8] (b)	[95] (m), [72] (p GC)	signaling molecule in ovarian ECM
**FMOD**	[1] (h); [14] (mo)	none	unknown
**LUM**	[1] (h); [14] (mo), [18] (p), [96] (b)	[97] (fetal b), [98] (CHO)	stromal expansion, cell migration, expression in CC
**PRELP**	[1] (h), [14] (mo),	none	unknown
**VCAN**	[18] (p)	[99](h serum), [100–102] (h CC), [103] (m, r)	binds hyaluronan, decreased in PCOS, CC, oocyte competency, development quality of oocytes

Abbreviations: b, bovine; c, canine; CC, cumulus cells; CHO, Chinese hamster ovarian cells; CL, corpus luteum; GC, granulosa cell; FF, follicular fluid; h, human; m, mouse; mo, monkey; o, ovine; p, porcine; PCOS, polycystic ovarian syndrome; r, rat; *, *see characterization*;

Supplemental References:

1. Ouni, E., Vertommen, D., Chiti, M., Dolmans, M.-M. & Amorim, C. A draft map of the human ovarian proteome for tissue engineering and clinical applications. *Mol Cell Proteomics* **18,** mcp.RA117.000469 (2018).

2. Marongiu, M. *et al.* Novel action of FOXL2 as mediator of Col1a2 gene autoregulation. *Dev Biol* **416,** 200 211 (2016).

3. Berkholtz, C. B., Lai, B. E., Woodruff, T. K. & Shea, L. D. Distribution of extracellular matrix proteins type I collagen, type IV collagen, fibronectin, and laminin in mouse folliculogenesis. *Histochem Cell Biol* **126,** 583–592 (2006).

4. Berkholtz, C., Shea, L. & Woodruff, T. Extracellular Matrix Functions in Follicle Maturation. *Semin Reprod Med* **24,** 262–269 (2006).

5. LIND, A. -K. *et al.* Collagens in the human ovary and their changes in the perifollicular stroma during ovulation. *Acta Obstet Gyn Scan* **85,** 1476–1484 (2006).

6. Figueiredo, J. R. *et al.* Extracellular matrix proteins and basement membrane: Their identification in bovine ovaries and significance for the attachment of cultured preantral follicles. *Theriogenology* **43,** 845–858 (1995).

7. Saha, S. *et al.* Localization and Thyroid Hormone Influenced Expression of Collagen II in Ovarian Tissue. *Cell Physiol Biochem* **19,** 67–76 (2007).

8. Hatzirodos, N. *et al.* Transcript abundance of stromal and thecal cell related genes during bovine ovarian development. *Plos One* **14,** e0213575 (2019).

9. Matti, N., Irving-Rodgers, H. F., Hatzirodos, N., Sullivan, T. R. & Rodgers, R. J. Differential expression of focimatrix and steroidogenic enzymes before size deviation during waves of follicular development in bovine ovarian follicles. *Mol Cell Endocrinol* **321,** 207 214 (2010).

10. Irving-Rodgers, H. F. *et al.* Dynamics of extracellular matrix in ovarian follicles and corpora lutea of mice. *Cell Tissue Res* **339,** 613 624 (2009).

11. Kranc, W. *et al.* Genes responsible for proliferation, differentiation, and junction adhesion are significantly up-regulated in human ovarian granulosa cells during a long-term primary in vitro culture. *Histochem Cell Biol* **151,** 125–143 (2019).

12. Hatzirodos, N., Hummitzsch, K., Irving-Rodgers, H. F. & Rodgers, R. J. Transcriptome Comparisons Identify New Cell Markers for Theca Interna and Granulosa Cells from Small and Large Antral Ovarian Follicles. *Plos One* **10,** e0119800 (2015).

13. Wang, L. *et al.* A two-dimensional electrophoresis reference map of human ovary. *J Mol Med* **83,** 812–821 (2005).

14. He, H. *et al.* Unravelling the proteome of adult rhesus monkey ovaries. *Mol Biosyst* **10,** 653 10 (2013).

15. Oksjoki, S., Sallinen, S., Vuorio, E. & Anttila, L. Cyclic expression of mRNA transcripts for connective tissue components in the mouse ovary. *Mhr Basic Sci Reproductive Medicine* **5,** 803–808 (1999).

16. Iwahashi, M., Muragaki, Y., Ooshima, A. & Nakano, R. Type VI collagen expression during growth of human ovarian follicles. *Fertil Steril* **74,** 343 347 (2000).

17. Li, X. *et al.* Dermatopontin is expressed in human liver and is downregulated in hepatocellular carcinoma. *Biochem Mosc* **74,** 979–985 (2009).

18. Hou, L., Wang, J., Wang, Y., Hua, X. & Wu, J. Compared proteomic analysis of 8- and 32-week-old postnatal porcine ovaries. *Cell Biochem Funct* **36,** 34–42 (2018).

19. Hassani, F. *et al.* Downregulation of Extracellular Matrix and Cell Adhesion Molecules in Cumulus Cells of Infertile Polycystic Ovary Syndrome Women with and without Insulin Resistance. *Cell J* **21,** 35–42

20. Chen, J., Wei, D., Zhao, Y., Liu, X. & Zhang, J. Overexpression of EFEMP1 Correlates with Tumor Progression and Poor Prognosis in Human Ovarian Carcinoma. *Plos One* **8,** e78783 (2013).

21. Bastian, N. A. *et al.* Regulation of fibrillins and modulators of TGFβ in fetal bovine and human ovaries. *Reproduction* **152,** 127–137 (2016).

22. Zhai, B. *et al.* BMP15 Prevents Cumulus Cell Apoptosis Through CCL2 and FBN1 in Porcine Ovaries. *Cell Physiol Biochem* **32,** 264–278 (2013).

23. Prodoehl, M. J. *et al.* Fibrillins and latent TGFβ binding proteins in bovine ovaries of offspring following high or low protein diets during pregnancy of dams. *Mol Cell Endocrinol* **307,** 133 141 (2009).

24. Prodoehl, M. J. *et al.* Genetic and gene expression analyses of the polycystic ovary syndrome candidate gene fibrillin-3 and other fibrillin family members in human ovaries. *Mol Hum Reprod* **15,** 829–41 (2009).

25. Kitasaka, H. *et al.* Inductions of granulosa cell luteinization and cumulus expansion are dependent on the fibronectin-integrin pathway during ovulation process in mice. *Plos One* **13,** e0192458 (2018).

26. Tamura, K., Yoshie, M., Hashimoto, K. & Tachikawa, E. Inhibitory effect of insulin-like growth factor-binding protein-7 (IGFBP7) on in vitro angiogenesis of vascular endothelial cells in the rat corpus luteum. *J Reprod Develop* **60,** 447–453 (2014).

27. Tamura, K., Matsushita, M., Endo, A., Kutsukake, M. & Kogo, H. Effect of Insulin-Like Growth Factor-Binding Protein 7 on Steroidogenesis in Granulosa Cells Derived from Equine Chorionic Gonadotropin-Primed Immature Rat Ovaries. *Biol Reprod* **77,** 485–491 (2007).

28. Ożegowska, K. *et al.* Genes Involved in the Processes of Cell Proliferation, Migration, Adhesion, and Tissue Development as New Potential Markers of Porcine Granulosa Cellular Processes In Vitro: A Microarray Approach. *Dna Cell Biol* **38,** 549–560 (2019).

29. Faraoni, E. Y. *et al.* Sex differences in the development of prolactinoma in mice overexpressing hCGβ: role of TGFβ1. *J Endocrinol* **232,** 535–546 (2017).

30. Dietzel, E. *et al.* Latent TGF-β binding protein-1 deficiency decreases female fertility. *Biochem Bioph Res Co* **482,** 1387–1392 (2017).

31. Kanai, Y. *et al.* Identification of a stromal cell type characterized by the secretion of a soluble integrin-binding protein, MFG-E8, in mouse early gonadogenesis. *Mech Develop* **96,** 223–227 (2000).

32. Mizukami, T. *et al.* Five azacytidine, a DNA methyltransferase inhibitor, specifically inhibits testicular cord formation and Sertoli cell differentiation in vitro. *Mol Reprod Dev* **75,** 1002–1010 (2008).

33. Naka, M. *et al.* Phagocytosis mechanism of apoptotic granulosa cells regulated by milk-fat globule-EGF factor 8. *Med Mol Morphol* **42,** 143–149 (2009).

34. Rosairo, D., Kuyznierewicz, I., Findlay, J. & Drummond, A. Transforming growth factor-β: its role in ovarian follicle development. *Reproduction* **136,** 799–809 (2008).

35. Pangas, S. A. Regulation of the ovarian reserve by members of the transforming growth factor beta family. *Mol Reprod Dev* **79,** 666–679 (2012).

36. Jackowska, M. *et al.* Differential expression of GDF9, TGFB1, TGFB2 and TGFB3 in porcine oocytes isolated from follicles of different size before and after culture in vitro. *Acta Vet Hung* **61,** 99–115 (2013).

37. Younis, A. J. *et al.* Extracellular-like matrices and leukaemia inhibitory factor for in vitro culture of human primordial follicles. *Reproduction Fertility Dev* **29,** 1982 (2017).

38. Carreiras, F. *et al.* Expression and Localization of αv Integrins and Their Ligand Vitronectin in Normal Ovarian Epithelium and in Ovarian Carcinoma. *Gynecol Oncol* **62,** 260–267 (1996).

39. Cruet, S., Salamanca, C., Mitchell, G. W. E. & Auersperg, N. αvβ3 and Vitronectin Expression by Normal Ovarian Surface Epithelial Cells: Role in Cell Adhesion and Cell Proliferation. *Gynecol Oncol* **75,** 254–260 (1999).

40. Jones, J. I., Doerr, M. E. & Clemmons, D. R. Cell migration: Interactions among integrins, IGFs and IGFBPs. *Prog Growth Factor Res* **6,** 319–327 (1995).

41. Mazerbourg, S., Zapf, J., Bar, R. S., Brigstock, D. R. & Monget, P. Insulin-Like Growth Factor (IGF)-Binding Protein-4 Proteolytic Degradation in Bovine, Equine, and Porcine Preovulatory Follicles: Regulation by IGFs and Heparin-Binding Domain-Containing Peptides. *Biol Reprod* **63,** 390–400 (2000).

42. Jia, Y. *et al.* Thiophenol-formaldehyde triazole causes apoptosis induction in ovary cancer cells and prevents tumor growth formation in mice model. *Eur J Med Chem* **172,** 62–70 (2019).

43. Nayaker, B. S. *et al.* Polycystic ovarian syndrome-associated cardiovascular complications: An overview of the association between the biochemical markers and potential strategies for their prevention and elimination. *Diabetes Metabolic Syndrome Clin Res Rev* **11,** S841–S851 (2017).

44. Cheng, Z. *et al.* Extracellular signal-regulated kinase 5 associates with casein kinase II to regulate GPIb-IX-mediated platelet activation via the PTEN/PI3K/Akt pathway. *J Thromb Haemost* **15,** 1679–1688 (2017).

45. Aziz, M. *et al.* Polycystic ovary syndrome: cardiovascular risk factors according to specific phenotypes. *Acta Obstet Gyn Scan* **94,** 1082–1089 (2015).

46. Shan, Y. *et al.* Coagulation and Fibrinolytic Indices During the First Trimester of Pregnancy in Women With Polycystic Ovary Syndrome. *Reprod Sci* **20,** 1390–1397 (2013).

47. Koiou, E. *et al.* Plasma Von Willebrand factor antigen levels are elevated in the classic phenotypes of polycystic ovary syndrome. *Hormones* **11,** 77–85 (2012).

48. Zhou, Z. *et al.* Novel mutations in ZP1, ZP2, and ZP3 cause female infertility due to abnormal zona pellucida formation. *Hum Genet* **138,** 327–337 (2019).

49. Canosa, S. *et al.* Zona pellucida gene mRNA expression in human oocytes is related to oocyte maturity, zona inner layer retardance and fertilization competence. *Mhr Basic Sci Reproductive Medicine* **23,** 292–303 (2017).

50. Gook, D. A., Edgar, D. H., Borg, J. & Martic, M. Detection of zona pellucida proteins during human folliculogenesis. *Hum Reprod* **23,** 394–402 (2008).

51. Kiefer, S. & Saling, P. Proteolytic Processing of Human Zona Pellucida Proteins1. *Biol Reprod* **66,** 407–414 (2002).

52. Greve, J. M., Salzmann, G. S., Roller, R. J. & Wassarman, P. M. Biosynthesis of the major zona pellucida glycoprotein secreted by oocytes during mammalian oogenesis. *Cell* **31,** 749–759 (1982).

53. Kempisty, B. *et al.* Expression and cellular distribution of zona pellucida glycoproteins in canine oocytes before and after in vitro maturation. *Zygote* **23,** 863–873 (2015).

54. Konrad, L. *et al.* Quantification of ZP1, ZP2 and ZP3 mRNA of marmoset monkey (Callithrix jacchus) oocytes from periantral and antral follicles. *Andrologia* **44,** 349–353 (2012).

55. Cariño, C. *et al.* Localization of species conserved zona pellucida antigens in mammalian ovaries. *Reprod Biomed Online* **4,** 116–126 (2002).

56. El-Mestrah, M., Castle, P. E., Borossa, G. & Kan, F. Subcellular Distribution of ZP1, ZP2, and ZP3 Glycoproteins During Folliculogenesis and Demonstration of Their Topographical Disposition Within the Zona Matrix of Mouse Ovarian Oocytes. *Biol Reprod* **66,** 866–876 (2002).

57. Rankin, T., Soyal, S. & Dean, J. The mouse zona pellucida: folliculogenesis, fertility and pre-implantation development. *Mol Cell Endocrinol* **163,** 21–25 (2000).

58. Haoula, Z. *et al.* Validation of proteomic biomarkers previously found to be differentially expressed in women with Polycystic Ovary Syndrome: a cross-sectional study. *Gynecol Endocrinol* **30,** 213–216 (2014).

59. Šunderić, M., Malenković, V. & Nedić, O. Complexes between insulin-like growth factor binding proteins and alpha-2-macroglobulin in patients with tumor. *Exp Mol Pathol* **98,** 173–177 (2015).

60. Zbroja-sontag, W. Defense Proteins and Immune Complexes in the Blood Serum of Women With Inflammatory and Neoplastic Lesions of the Ovary. *Am J Reprod Immunol* **4,** 11–20 (1983).

61. ee, Fey, G. & Richards, J. Stat 5b and the orphan nuclear receptors regulate expression of the alpha2-macroglobulin (alpha2M) gene in rat ovarian granulosa cells. *Mol Endocrinol Baltim Md* **12,** 1393–409 (1998).

62. Hamm, A. *et al.* Frequent expression loss of Inter-alpha-trypsin inhibitor heavy chain (ITIH) genes in multiple human solid tumors: A systematic expression analysis. *Bmc Cancer* **8,** 25 (2008).

63. Falfushynska, H., Gnatyshyna, L., Deneha, H., Osadchuk, O. & Stoliar, O. MANIFESTATIONS OF OXIDATIVE STRESS AND MOLECULAR DAMAGES IN OVARIAN CANCER TISSUE. *Ukrainian Biochem J* **87,** 93–102 (2015).

64. Aboelenain, M. *et al.* Status of autophagy, lysosome activity and apoptosis during corpus luteum regression in cattle. *J Reprod Develop* **61,** 229–236 (2015).

65. Kakoly, N., Earnest, A., Moran, L. J., Teede, H. J. & Joham, A. E. Group-based developmental BMI trajectories, polycystic ovary syndrome, and gestational diabetes: a community-based longitudinal study. *Bmc Med* **15,** 195 (2017).

66. Cai, L. *et al.* The effects of human recombinant granulocyte-colony stimulating factor treatment during in vitro maturation of porcine oocyte on subsequent embryonic development. *Theriogenology* **84,** 1075–1087 (2015).

67. Nordqvist, S. *et al.* The Presence of Histidine-Rich Glycoprotein in the Female Reproductive Tract and in Embryos. *Reprod Sci* **17,** 941–947 (2010).

68. Xu, F. *et al.* The outcome of heregulin-induced activation of ovarian cancer cells depends on the relative levels of HER-2 and HER-3 expression. *Clin Cancer Res Official J Am Assoc Cancer Res* **5,** 3653–60 (1999).

69. Zachut, M., Sood, P., Levin, Y. & Moallem, U. Proteomic analysis of preovulatory follicular fluid reveals differentially abundant proteins in less fertile dairy cows. *J Proteomics* **139,** 122–129 (2016).

70. Ilha, G. *et al.* Characterization of the kallikrein–kinin system during the bovine ovulation process. *Peptides* **32,** 2122–2126 (2011).

71. Zheng, Q. *et al.* Novel Serum Biomarkers Detected by Protein Array in Polycystic Ovary Syndrome with Low Progesterone Level. *Cell Physiol Biochem* **46,** 2297–2310 (2018).

72. Chermuła, B. *et al.* New Gene Markers of Angiogenesis and Blood Vessels Development in Porcine Ovarian Granulosa Cells during Short-Term Primary Culture In Vitro. *Biomed Res Int* **2019,** 1 12 (2019).

73. Zhang, C., Ma, J., Wang, W., Sun, Y. & Sun, K. Lysyl oxidase blockade ameliorates anovulation in polycystic ovary syndrome. *Hum Reprod* **33,** 2096–2106 (2018).

74. Chang, H.-M. *et al.* Activin A-induced increase in LOX activity in human granulosa–lutein cells is mediated by CTGF. *Reproduction* **152,** 293–301 (2016).

75. Fang, Y. *et al.* Transforming growth factor-β1 increases lysyl oxidase expression by downregulating MIR29A in human granulosa lutein cells. *Reproduction* **152,** 205–213 (2016).

76. Weitzel, J., Vernunft, A., Krüger, B., Plinski, C. & Viergutz, T. LOX-1 regulates estrogenesis via intracellular calcium release from bovine granulosa cells. *Cytom Part A* **85,** 88–93 (2014).

77. Weitzel, J. M., Vernunft, A., Krüger, B., Plinski, C. & Viergutz, T. Inactivation of the LOX-1 Pathway Promotes the Golgi Apparatus during Cell Differentiation of Mural Granulosa Cells. *J Cell Physiol* **229,** 1946–1951 (2014).

78. Löhrke, B. *et al.* Lectin-like oxidized low-density lipoprotein receptor-1 (LOX-1) activity decreases estrogenesis in ovarian granulosa cells. *Cytom Part A* **79A,** 669–671 (2011).

79. Harlow, C. R., Rae, M., Davidson, L., Trackman, P. C. & Hillier, S. G. Lysyl oxidase gene expression and enzyme activity in the rat ovary: regulation by follicle-stimulating hormone, androgen, and transforming growth factor-beta superfamily members in vitro. *Endocrinology* **144,** 154–62 (2003).

80. Burchall, G. F. *et al.* Expression of the plasminogen system in the physiological mouse ovary and in the pathological polycystic ovary syndrome (PCOS) state. *Reprod Biol Endocrin* **17,** 33 (2019).

81. Patil, K. *et al.* Quantitative mass spectrometric analysis to unravel glycoproteomic signature of follicular fluid in women with polycystic ovary syndrome. *Plos One* **14,** e0214742 (2019).

82. Kaur, S. *et al.* Differential Gene Expression in Granulosa Cells from Polycystic Ovary Syndrome Patients with and without Insulin Resistance: Identification of Susceptibility Gene Sets through Network Analysis. *J Clin Endocrinol Metabolism* **97,** E2016–E2021 (2012).

83. Huang, Y. *et al.* Discovery of serum biomarkers implicated in the onset and progression of serous ovarian cancer in a rat model using iTRAQ technique. *Eur J Obstet Gyn R B* **165,** 96–103 (2012).

84. Douville, G. & Sirard, M.-A. Changes in granulosa cells gene expression associated with growth, plateau and atretic phases in medium bovine follicles. *J Ovarian Res* **7,** 50 (2014).

85. Matsuzaki, S. *et al.* Anti-glypican-1 antibody-drug conjugate exhibits potent preclinical antitumor activity against glypican-1 positive uterine cervical cancer. *Int J Cancer* **142,** 1056–1066 (2018).

86. TUNGK, D. *et al.* Effects of gonadotropin-releasing hormone agonist on human chorionic gonadotropin activity in granulosa cells of immature female rats. *J Reprod Develop* **64,** 2017–142 (2017).

87. Hatzirodos, N. *et al.* Transcript abundance of stromal and thecal cell related genes during bovine ovarian development. *Plos One* **14,** e0213575 16 (2019).

88. Nio-Kobayashi, J. & Iwanaga, T. Differential Cellular Localization of Galectin-1 and Galectin-3 in the Regressing Corpus Luteum of Mice and Their Possible Contribution to Luteal Cell Elimination. *J Histochem Cytochem* **58,** 741–749 (2010).

89. Nio, J. & Iwanaga, T. Galectins in the Mouse Ovary: Concomitant Expression of Galectin-3 and Progesterone Degradation Enzyme (20α-HSD) in the Corpus Luteum. *J Histochem Cytochem* **55,** 423–432 (2007).

90. Chen, L. *et al.* Clinical implication of the serum galectin-1 expression in epithelial ovarian cancer patients. *J Ovarian Res* **8,** 78 (2015).

91. Sano, M., Hashi, K., Nio-Koyashi, J. & Okuda, K. The luteotrophic function of galectin-1 by binding to the glycans on vascular endothelial growth factor receptor-2 in bovine luteal cells. *J Reprod Develop* **61,** 439–448 (2015).

92. Walzel, H. *et al.* Effects of galectin-1 on regulation of progesterone production in granulosa cells from pig ovaries in vitro. *Glycobiology* **14,** 871–881 (2004).

93. Dhali, A. *et al.* Temporal expression of cumulus cell marker genes during in vitro maturation and oocyte developmental competence. *J Assist Reprod Gen* **34,** 1493–1500 (2017).

94. Davies, J. E. *et al.* Distribution and Clinical Significance of Heparan Sulfate Proteoglycans in Ovarian Cancer. *Clin Cancer Res* **10,** 5178–5186 (2004).

95. Adam, M. *et al.* Decorin is a part of the ovarian extracellular matrix in primates and may act as a signaling molecule. *Hum Reprod* **27,** 3249–3258 (2012).

96. Bunel, A. *et al.* Individual bovine in vitro embryo production and cumulus cell transcriptomic analysis to distinguish cumulus-oocyte complexes with high or low developmental potential. *Theriogenology* **83,** 228–237 (2015).

97. Hartanti, M. *et al.* Morphometric and gene expression analyses of stromal expansion during development of the bovine fetal ovary. *Reproduction Fertility Dev* **31,** 482 (2018).

98. Zeltz, C. *et al.* Lumican inhibits cell migration through α2β1 integrin. *Exp Cell Res* **316,** 2922–2931 (2010).

99. Özler, S. *et al.* Role of Versican and ADAMTS-1 in Polycystic Ovary Syndrome. *J Clin Res Pediatr E* **9,** 24–30 (2017).

100. Ocampo, A. *et al.* Assessment of Prostaglandin-Endoperoxide Synthase 2 and Versican gene expression profile from the cumulus cells: association with better in vitro fertilization outcomes. *J Ovarian Res* **11,** 84 (2018).

101. Liu, Q. *et al.* Analyzing the Transcriptome Profile of Human Cumulus Cells Related to Embryo Quality via RNA Sequencing. *Biomed Res Int* **2018,** 1–8 (2018).

102. Adriaenssens, T. *et al.* Cumulus cell gene expression is associated with oocyte developmental quality and influenced by patient and treatment characteristics. *Hum Reprod* **25,** 1259–1270 (2010).

103. Russell, D. L., Ochsner, S. A., Hsieh, M., Mulders, S. & Richards, J. S. Hormone-regulated expression and localization of versican in the rodent ovary. *Endocrinology* **144,** 1020–31 (2003).
